# African voices and leadership is imperative for the global AIDS response

**DOI:** 10.11604/pamj.2019.32.87.18452

**Published:** 2019-02-22

**Authors:** Francois-Xavier Mbopi-Keou, Brian Williams, Laurent Bélec, Ginette Claude Mireille Kalla, Ibra Ndoye, Mamadou Henri Konate, Tina Gifty Mensah

**Affiliations:** 1 University of Yaoundé I, Faculty of Medicine and Biomedical Sciences, Yaoundé, Cameroon; 2 The Institute for the Development of Africa (The-IDA), Yaoundé, Cameroon; 3 South Africa Centre for Epidemiological Modelling and Analysis, University of Stellenbosch, Stellenbosch, South Africa; 4 Hôpital Européen Georges Pompidou, Université Paris Descartes, Paris Sorbonne Cité, France; 5 Coordinating Board of CRCF and ANRS Research Site in Senegal, Dakar, Senegal; 6 Embassy of Mali & Mali Permanent Mission to the United Nations Office and other International Organizations in Geneva, Geneva, Switzerland; 7 Parliament of Ghana & Ministry of Health, Accra, Ghana

**Keywords:** African voices, leadership, global AIDS response, opinion

## Abstract

This position paper is written in reference to the recent extensive media coverage of the report of the Independent Panel describing Harassment, Including Sexual Harassment, Bullying and Abuse of Power at UNAIDS Secretariat by several newspapers and authoritative journals such as *Science* and *The Lancet*. Unfortunately, none of these publications provide any clear evidence to support the accusations and merely repeat what are, in our view, unsubstantiated statements made in the report. Given the critical role that Africans have played in dealing with one of the most severe epidemics that the world has seen and the gravity of these charges, we believe it is essential to reaffirm that African voices and leadership is imperative for the global AIDS response.

## Opinion

This position paper is written in reference to the recent extensive media coverage of the report of the Independent Panel describing Harassment, Including Sexual Harassment, Bullying and Abuse of Power at UNAIDS Secretariat [1] by several newspapers and authoritative journals such as *Science* and *The Lancet*.

The African continent and its response to the AIDS epidemic are very different from what they were ten years ago. In spite of the fact that African countries have experienced some of the worst epidemics of HIV in the world, African leadership has been critical in the response to the epidemic of HIV leading to an unprecedented reduction in AIDS-related deaths and greatly reduced new HIV infections. While Africa still bears the brunt of the epidemic, it has also had the largest share of success with lessons for all the other affected countries. UNAIDS has been instrumental in that transformation, facilitating policy change, holding the lamp for human rights, creating a new discourse on access to life saving medicines and addressing stigma and discrimination while ensuring accountability and sustainability.

The many successes in dealing with HIV in Africa have been achieved by the good work of many people but led by Michel Sidibé, a charismatic and visionary son of Africa. Now UNAIDS is under the spotlight and has had to look carefully to itself in order to deal with problems that inevitably arise in such a large organization and to ensure a safe and secure working environment for all its staff [2-4]. Only a leader with courage would call for an independent expert panel to examine the organization, its structure and its practice and provide a better path to the future. A leader who has the courage to do that will surely have the courage to implement the changes that are needed. One only has to look at this history of the bold changes Sidibé has brought to UNAIDS. In 2011, he set a target to double the number of people on treatment; ‘15 by 15’ or 15 million people on ART by 2015 was set and achieved well ahead of the deadline. Then he led the world in setting and working to achieve the transformative vision of the 90-90-90 target for the percentage of people that know their status, of these that are on ART and of these that are virally suppressed. Only Sidibé could challenge and persuade President Zuma to transform South Africa’s AIDS response, remove protectionism and convince his government to spend US$ 2 billion annually, year after year, and effectively bring under control the biggest epidemic in the world. While many African countries still have discriminatory laws and often ignore the rights of key populations, Sidibé has spoken to their leaders, challenged them, and the dialogue has begun. There are few in global health who can look presidents and prime ministers in the eye and demand investments and changes in policy with courage and conviction as Sidibé has done. Within UNAIDS the staff have benefited from and appreciated his open and supportive management with a human face.

UNAIDS is more diverse than any other UN organization and is committed to equality for all staff in all their diversity. He has restructured UNAIDS several times in order to make it fit for purpose and to meet the challenges and the mandate the global community gave it. Through his actions and leadership hundreds of members of staff have retained their jobs in times of grave financial crisis and many more women have been promoted than in previous years. While he may not have acted on issues of harassment decisively and quickly enough as suggested by the report of the Independent Panel this does not mean that he has been insensitive to these issues, has not heard these criticisms or does not possess the skills needed to turn the institution around.

In the future, UNAIDS could be led by a woman who could carry on the great legacy of Michel Sidibé. A woman leader would be well placed to confront Africa and the larger world on issues of violence against women, the harmful social norms that disadvantage women, in Africa such as anywhere and leverage the growing power of the women’s movement to bring about change.

## Competing interests

The authors declare no competing interests.
